# Visual alertness and brain diffusion tensor imaging at term age predict neurocognitive development at preschool age in extremely preterm‐born children

**DOI:** 10.1002/brb3.3048

**Published:** 2023-05-10

**Authors:** Leena Aho, Viljami Sairanen, Piia Lönnberg, Elina Wolford, Aulikki Lano, Marjo Metsäranta

**Affiliations:** ^1^ New Children's Hospital, Pediatric Research Center University of Helsinki and Helsinki University Hospital Helsinki Finland; ^2^ BABA Center, Pediatric Research Center, Department of Clinical Neurophysiology Children's Hospital Helsinki University Hospital and University of Helsinki Helsinki Finland; ^3^ Department of Psychology and Logopedics University of Helsinki Helsinki Finland

**Keywords:** diffusion tensor imaging, neurodevelopment, preterm birth, social cognition, visual alertness

## Abstract

**Introduction:**

Cognitive development is characterized by the structural and functional maturation of the brain. Diffusion‐weighted magnetic resonance imaging (dMRI) provides methods of investigating the brain structure and connectivity and their correlations with the neurocognitive outcome. Our aim was to examine the relationship between early visual abilities, brain white matter structures, and the later neurocognitive outcome.

**Methods:**

This study included 20 infants who were born before 28 gestational weeks and followed until the age of 6.5 years. At term age, visual alertness was evaluated and dMRI was used to investigate the brain white matter structure using fractional anisotropy (FA) in tract‐based spatial statistics analysis. The JHU DTI white matter atlas was used to locate the findings. The neuropsychological assessment was used to assess neurocognitive performance at 6.5 years.

**Results:**

Optimal visual alertness at term age was significantly associated with better visuospatial processing (*p* < .05), sensorimotor functioning (*p* < .05), and social perception (*p* < .05) at 6.5 years of age. Optimal visual alertness related to higher FA values, and further, the FA values positively correlated with the neurocognitive outcome. The tract‐based spatial differences in FA values were detected between children with optimal and nonoptimal visual alertness according to performance at 6.5 years.

**Conclusion:**

We provide neurobiological evidence for the global and tract‐based spatial differences in the white matter maturation between extremely preterm children with optimal and nonoptimal visual alertness at term age and a link between white matter maturation, visual alertness and the neurocognitive outcome at 6.5 years proposing that early visual function is a building block for the later neurocognitive development.

## Graphical abstract

The relationship between early visual abilities, brain white matter structures, and later neurocognitive outcome in extremely preterm born infants was examined. At term age, visual alertness was evaluated and dMRI was used to investigate the brain white matter structure and at 6.5 years neurocognitive performance was assessed. Optimal visual alertness related to widely increased mean fractional anisotropy (FA), and the mean FA values positively correlated with neurocognitive outcome proposing that early visual function is a building block for the later neurocognitive development.

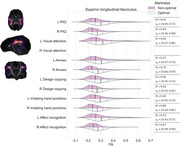

## 1 INTRODUCTION

Extremely preterm (EPT) born children are at a high risk for neurodevelopmental impairments. In follow‐up studies from 25% to 50% of EPT children have cognitive, motor, behavioral, and social impairments that may affect their life‐course opportunities (Geldof et al., 2012; Johnson & Marlow, 2014). Cognitive development is characterized by the structural and functional maturation of the brain and preterm birth may interrupt this normal maturation and poses a risk for neurocognitive impairment.

Neurocognitive development can be described as a cognitive cascade where few basic abilities are the foundation of later cognition (Rose et al., 2008). Brain growth and maturation are asynchronous, sensory pathways develop early and quickly, whereas associative regions develop later and slowly (Huang et al., 2006). Sensory attention, especially vision, is one of the earliest fundamental brain mechanisms acquired. EPT children are at a high risk for cerebral visual impairments, up to 48% of EPT children without ophthalmological impairments have visual processing problems (Pel et al., 2016), which reflect the encephalopathy of prematurity including diffuse white matter injury (Back, 2017). The white matter and periventricular injury may lead to damage in the optic radiations, the tracts that convey visual information from the lateral geniculate nucleus to the primary visual cortex (Dutton & Jacobson, 2001). Such damage in the visual pathways could be at the microstructural level and remain undetected by conventional magnetic resonance imaging (Pel et al., 2016).

We have earlier demonstrated in our longitudinal prospective cohort study that the good visual fixation with sustained alertness at term age was significantly related to visuomotor performance at 2 and 5 years of age. In addition, the good visual sustained fixation, when tracking an object, related to widely increased fractional anisotropy (FA) values across the white matter at term age (Stjerna et al., 2015). This suggests for structural differences in the subcortical networks, which are fundamental for the earliest visual fixation and attention.

Moreover, visual processing deficits may result in difficulties to identify key nonverbal cues from facial and body movements among very low birth weight children leading to social behavior impairments (Williamson & Jakobson, 2014). We have also shown using the same cohort that optimal visual alertness at term age was associated with the better development of social cognition skills at preschool age (Aho et al., 2021).

In vivo investigation of the brain white matter structures and their possible correlations with neurocognitive test results can be achieved using diffusion‐weighted magnetic resonance imaging. dMRI provides methods for indirect evaluation of the brain microstructure and its integrity as well as structural connectivity of the brain (Basser and Pierpaoli, 1996). Previous studies have demonstrated that dMRI can reveal associations between parameters derived from diffusion tensor model (Basser et al., 1994) and neurobehavioral outcome (Arzoumanian et al., 2003; Stjerna et al., 2015; Weinstein et al., 2016).

In this study, we argue that visual alertness and the brain white matter structures investigated using dMRI at term age is associated with the neurocognitive outcome and social cognition skills at preschool age. We test this hypothesis with thorough statistical analysis on both neurocognitive tests and diffusion tensor derived FA values. In the future, such FA‐based correlation could be beneficial for providing individualized treatment and interventions at the early stage of EPT children's life.

## 2 MATERIALS AND METHODS

### 2.1 Participants

**FIGURE 1:**
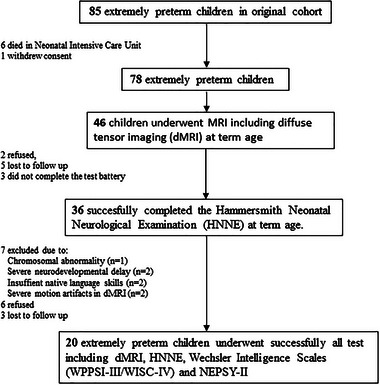
Study flowcharts and dropouts.

The original cohort of this longitudinal multimethodological prospective study included 85 EPT infants who were born before 28 gestational weeks and were actively treated after birth at the neonatal intensive care unit of the Helsinki University Hospital, Finland between May 2006, and September 2008 (KeKeKe study—Extremely Preterm Birth and Development of the Central Nervous System). The present study included 22 EPT children who had been successfully followed until 6.5 years of age and undergone all tests reported here (Figure 1). The magnetic resonance imaging was performed for 70 EPT infants of which 46 were scanned also with diffusion‐weighted sequences. Rest of the infants (24) were too eager to move and dMRI could not be acquired from them. In total, 22 EPT born children underwent successfully all tests including dMRI, the Hammersmith Neonatal Neurological Assessment (HNNE) and the neuropsychological assessment at 6.5 years of age. Two children were later rejected due to having severe motion artifacts in dMRI measurements.

The original study and the follow‐up at 6.5 years were approved by The Ethics Committee for gynecology and obstetrics, pediatrics, and psychiatry of the Hospital District of Helsinki and Uusimaa. The written informed consent to participation and publication of the results was obtained from the parents and guardians. In addition, all children received age‐appropriate information about the study and provided consent.

### 2.2 Clinical data

Obstetric and neonatal data were collected from the medical records and are presented in Table 1. Socioeconomical data were obtained from parental questionnaires. Gestational age was based on the first‐trimester ultrasound. The small for gestational age was defined as a birth weight below –2.0 SD of the gestational age from the Finnish growth reference data. Bronchopulmonary dysplasia was defined as a need for additional oxygen at 36 + 0 weeks of gestational age. Retinopathy of prematurity was diagnosed by an ophthalmologist at routine visits. Severe retinopathy was defined as ROP Stage III treated with retinal laser photocoagulation. Patent ductus arteriosus was determined by cardiac ultrasound. The highest grade of intraventricular hemorrhage in serial cranial ultrasound was recorded. MRI was performed at term age. White matter injury was classified into four categories from none to severe (Woodward et al., 2006).

**TABLE 1:** Characteristics of study population and dropouts

	Dropouts	Participants		Participants		
				Visual alertness		
	*n* = 58	*n* = 20	*p*	Optimal *n* = 10	Nonoptimal *n* = 10	*p*
Boys, *n* (%)	35 (60)	14 (70)	0.662	6 (60)	8 (80)	.628
Gestational age, mean (SD)	26 (1.2)	27 (1.1)	0.356	26 (1.1)	27 (1.1)	.522
Birth weight, g mean (SD)	858 (174)	895 (190)	0.328	866 (222)	924 (158)	.505
Twins, *n* (%)	7 (12)	9 (45)	0.003	5 (50)	4 (40)	1.0
SGA, *n* (%)	7 (12)	2 (10)	1.0	1 (10)	1 (10)	.763
Neonatal morbidity, *n* (%)						
Prenatal corticosteroids	58 (100)	19 (95)	0.256	10 (100)	9 (90)	1.0
Respiratory distress syndrome	36 (62)	19 (95)	0.004	10 (100)	9 (90)	1.0
Bronchopulmonary dysplasia at 36+0 gestation weeks	28 (48)	6 (30)	0.281	4 (40)	2 (20)	.638
Necrotizing enterocolitis	6 (2)	2 (10)	0.828	0	2 (20)	.474
Patent ductus arteriosus	46 (79)	16 (80)	1.0	8 (80)	8 (80)	.856
Sepsis, blood stream positive	27 (47)	8 (40)	0.437	3 (30)	5 (50)	.650
Severe retinopathy of prematurity	19 (33)	4 (20)	0.771	3 (30)	1 (10)	.582
Intraventricular hemorrhage, *n*			0.154			.484
No	39 (67)	9 (45)		6 (60)	3 (30)	
Grade I–II	10 (17)	8 (40)		3 (30)	5 (50)	
Grade III–IV	9 (16)	3 (15)		1 (10)	2 (20)	
White matter injury in MRI at term age			0.197			.395
Normal	34 (59)	10 (50)		5 (50)	5 (50)	
Mild	12 (21)	8 (40)		5 (50)	3 (30)	
Moderate	4 (7)	2 (10)			2 (20)	
Severe						
Mother's education, *n*			0.122			.060
High school or lower	25 (49)	6 (30)		1 (10)	5 (50)	
Bachelor's degree	15 (29)	7 (35)		4 (40)	3 (30)	
Master's degree or higher	11 (22)	7 (35)		5 (50)	2 (20)	

Data not available for MRI for 8 dropouts and for mother's education for 7 dropouts and for bronchopulmonary dysplasia for two EPT children with nonoptimal visual alertness. SGA (small for gestation age) is defined as a birth weight below –2.0 SD.

Fischer's exact, Mann–Whitney *U*, and Kendall tau *B* test were used.

### 2.3 Neonatal neurological assessment and neuropsychological assessment at 6.5 years of age

The neonatal neurological examination was performed by an experienced pediatric neurologist (AL) at term age using the standardized HNNE (Dubowitz et al., 1998). Visual alertness was assessed with standard black and white bull's eye target and the best performance was recorded (Dubowitz et al., 1998). We used dichotomic rating, optimal and nonoptimal. The performance was defined as optimal if infant keeps interest in stimuli.

A neuropsychological assessment was conducted at 6.5 years of age. General cognitive development was assessed using the Finnish edition of the Wechsler Preschool and Primary Scale of Intelligence—Third Edition (WPPSI‐III) or the Wechsler Intelligence Scale for Children—Fourth Edition (WISC‐IV). Full Scale (FSIQ), Verbal (VIQ), and Performance Intelligence Quotients (PIQ; mean 100, SD 15) were derived from three Performance (Block Design, Matrix Reasoning, and Picture Completion) and two Verbal (Information and Vocabulary) subtests of the WPPSI‐III or the WISC‐IV. Neuropsychological functioning was assessed with the Finnish edition of NEPSY‐II, a neuropsychological test battery (Korkman et al., 2008a; Wechsler, 2009, 2010). Five subtests were used to test different domains of neuropsychological functioning and social perception: executive functioning/attention (Visual attention), sensorimotor functioning (Imitating hand positions), visuospatial processing (Arrows, Design copying), and social perception (Affect recognition). Social perception test assesses social cognition skills. Age‐adjusted standard scores for the subtests were calculated according to the Finnish norms (mean 10, SD 3) (Korkman et al., 2008b).

### 2.4 Magnetic resonance imaging

Whole brain diffusion‐weighted MRI scans were performed using a Philips Achieva 1.5T scanner (Best, Netherlands) with an 8‐channel phased array head coil. A single‐shot echo planar imaging (EPI) sequence was used to obtain one nondiffusion weighted image and 15 diffusion‐weighted images using the over‐plus gradient scheme provided by the scanner vendor. The scan was repeated three times for each subject to obtain redundancy for lost data due to subject motion and to obtain more data for reliable tensor model estimation. All three scans were concatenated into a set of three reference images and 45 diffusion‐weighted images. No averaging was done. Scan parameters were a repetition time: shortest in the range from 6085 to 6778 ms, an echo time: 58 ms, a voxel size 1.75 × 1.75 × 2 mm^3^ with an imaging matrix of 128 × 128, 44 axial slices with no gap between them, a slice thickness of 2 mm, no averaging of the measurements, and an in‐plane acceleration (SENSE) 2. The used *b*‐value for diffusion weighting was 700 mm/s^2^.

### 2.5 MRI analysis

Diffusion‐weighted images were processed using ExploreDTI (Leemans et al., 2009) accompanied by SOLID‐plugin (Sairanen et al., 2018 A) to adjust for subject motion related artifacts simultaneously with the subject motion and eddy current distortion corrections. Prior to the motion correction, a correction for Gibbs ringing was performed (Perrone et al., 2015). Signal drift was not observed in dMRI data therefore it was not corrected for (Vos et al., 2017). However, some of the subjects had woken up during the scanning and some of them required repositioning and reshimming between the three diffusion‐weighted scans. Therefore, the shimming might have been slightly different between the repeated three dMRI acquisitions as the patient head might have been in different orientation and location. To adjust for this, we implemented a simple normalization of the affected images by scaling the intensities to match between nondiffusion‐weighted images and applied the correction factor to diffusion weighted images too using an approach very similar to the signal drift correction (Vos et al., 2017).

The robust wild residual bootstrapping (Sairanen et al., 2018 B) of the motion corrected images with outlier information (Sairanen et al., 2018 A) was used to obtain estimates and confidence intervals for the voxelwise diffusion tensor distributions. In total, 2000 bootstrap samples were calculated to obtain these distributions. Based on the robust bootstrapping confidence intervals, FA could be correctly estimated whereas mean diffusivity (MD) did not converge to specific values in the brain white matter. This was likely due to too sparse sampling of the *q*‐space with the used 15‐direction over‐plus gradient scheme (Jones, 2004). However, three repetitions of the scan were enough to produce reliable median FA estimates into which we limited our investigations.

Median FA images calculated from the bootstrap samples were processed using tract‐based spatial statistics (TBSS) (Bach et al., 2014; Smith et al., 2006) pipeline implemented in FSL program 6.03 (Smith et al., 2004). A study specific target was obtained by cross‐registering every subject to each other as registering infant brain images directly to the default MNI152 adult brain atlas might not provide accurate results. FA skeletons resulting from TBSS were analyzed using randomize program (Smith & Nichols, 2009; Winkler et al., 2014) implemented in FSL to perform nonparametric statistical tests with 10,000 permutations. With TBSS we evaluated a simple linear model to study the correlation between FA values and the neurocognitive test scores in the visually alert optimal and nonoptimal groups. We controlled the performed multiple comparisons using Bonferroni correction. We used significance level of .05 to indicate statistically significant findings.

Due to the used 15‐direction dMRI protocol with three repetitions, tractography could not be performed reliably. Therefore, we used white matter streamline atlas (JHU DTI‐based white matter atlas) (Hua et al., 2008; Mori et al., 2005; Wakana et al., 2007) to approximate the locations and shapes of the corresponding white matter structures. This approximation allowed us to pinpoint the statistically significant findings of TBSS to specific white matter bundles without performing tractography on the suboptimal data. Streamline atlas was used only to mask the TBSS results and this step did not include any additional statistical tests.

Effect sizes, that is, differences in FA values, in the regions where TBSS resulted statistically significant findings, were further visualized using violin plots for each white matter streamline bundle separately. We evaluated the differences between these violins using Cohen's *D* and Akinshin's gamma effect size estimates. The Cohen's *D* reports the average difference between the groups whereas Akinshin's gamma reports a range from minimal and maximal robust differences which should be more suitable for nonnormal distributions. The streamline atlas consists of following monolateral white matter bundles: anterior thalamic radiation (ATR), corticospinal tract (CST), cingulum cingulate gyrus (CGY), cingulum hippocampus (CHI), inferior frontooccipital fasciculus (IFOF), inferior longitudinal fasciculus (ILF), superior longitudinal fasciculus (SLF), uncinate fasciculus (UF), and temporal part of the superior longitudinal fasciculus (SLF_temp) as well as bilateral white matter bundles: forceps major (FMA) and forceps minor (FMI). With these segments, we were able to approximate the locations of the white matter differences between the groups.

### 2.6 Statistical analysis of clinical data and neuropsychological assessments

Statistical analysis was conducted using SPSS for Windows program version 27. Background variables between children with optimal and nonoptimal alertness were compared using Fischer's exact test and Kendall tau *B* test for categorical variables and Mann–Whitney *U* test for continuous variables. The relationship between visual alertness and IQs in WPPSI‐III/WISC‐IV and standard scores in NEPSY were examined using linear regression analysis. Results are reported as coefficients β and their 95% confidence interval (CI). The distribution of residuals was assessed visually from histograms and by Shapiro–Wilk test. All significance tests were two‐tailed and *p* < .05 was considered significant.

## 3 RESULTS

The characteristics of study population and dropouts are shown in Table 1. None of the participating EPT children were blind, deaf, or had cerebral palsy. Half of the participating EPT infants (*n* = 10) showed optimal visual alertness at term age. The sex or neonatal morbidity factors (Table 1) were not associated with the performance on visual alertness.

### 3.1 Neuropsychological outcome at 6.5 years

The mean FSIQ, VIQ, and PIQ corresponded to the average for Finnish children according to test norms (Table 2). The standard scores for NEPSY‐II subtests Visual attention, Arrows, and Affect recognition were in the average range (8–12). The standard scores in Imitating hand positions and Design copying were borderline (7.0, SD 3.4, and 7.5, SD 2.1, respectively). The only sex difference occurred in Arrows, girls scored 3.4 standard scores less than boys (95% CI –5.5 to –1.3, *p* = .003). Table 2 shows the associations between visual alertness and neuropsychological outcome at 6.5 years. EPT infants with optimal visual alertness achieved 3.3 standard scores more in sensorimotor function test, Imitating hand positions (*p* = .025, 95% CI 0.5–6.1), 2.6 standard scores more in visuospatial processing test, Arrows (*p* = .018, 95% CI 0.5–4.7) and 2.6 standard scores more in social perception test, Affect recognition (*p =* .012, 95% CI 0.6–4.6). The estimated difference was clinically notable, approximately 1 SD (SD is 3 standard score).

**TABLE 2:** The association between visual alertness and outcomes at 6.5 years

	All	Visual alertness				
	*n* = 20	Optimal *n* = 10	Nonoptimal *n* = 10			
	Mean (SD)	Mean (SD)	Mean (SD)	β	95% Cl	*p*
**WPPSI‐III / WISC‐IV, IQ** (mean 100, SD 15)						
FSIQ	98 (8.7)	98 (11.6)	98 (5.1)	0.7	−7.6 to 9.1	.868
VIQ	107 (10.5)	106 (12.5)	108 (8.5)	2.1	−12.1 to 7.9	.666
PIQ	89 (12.3)	91 (15.1)	87 (9.1)	3.5	−8.2 to 15.2	.539
**NEPSY‐II, standard score** (mean 10, SD 3)						
Executive function/attention						
Visual attention	9.7 (1.8)	10.0 (1.6)	9.3 (2.0)	0.7	−1.1 to 2.4	.405
Visuospatial processing						
Arrows	9.5 (2.5)	10.8 (2.1)	8.2 (2.3)	2.6	0.51 to 4.7	.018
Design copying	7.5 (2.1)	8.2 (2.7)	6.8 (0.9)	1.4	−0.5 to 3.3	.138
Sensorimotor function						
Imitating hand positions	7.0 (3.4)	8.6 (3.3)	5.3 (2.7)	3.3	0.46 to 6.1	.025
Social perception						
Affect recognition	8.7 (2.4)	10.0 (1.7)	7.4 (2.4)	2.6	0.64 to 46	.012

The unadjusted linear regression model was performed. β‐estimated mean difference was performed.

WPPSI‐III, Wechsler Preschool and Primary Scale of Intelligence—Third Edition; WISC, Wechsler Intelligence Scale for Children—Fourth Edition; FSIQ, full scale; VIQ, verbal; PIQ, Performance Intelligence Quotients; NEPSY‐II, a Developmental Neuropsychological Assessment.

### 3.2 Visual alertness is associated with changes in white matter microstructure and later neurocognitive outcome

The associations between visual alertness, the FA values in different white matter bundles at term age and neuropsychological performance at preschool age are shown in Figure 2. TBSS demonstrated a significant linear correlation between the FA values in the different white matter bundles at term age and neuropsychological performance at 6.5 years of age (nonparametric permutation test *p* < .05). The estimated effect sizes between children with optimal and nonoptimal visual alertness in different white matter bundles are given in Figure 2 and Table 3.

FIGURE 2FA values of the white matter correlated with performance IQ and each neuropsychological subtests among preterm children with optimal and nonoptimal visual alertness. Each subfigure presents the overlap of white matter region with (a) corticospinal tract, (b) cingulum cingulate gyrus, (c) cingulum hippocampus, (d) inferior frontooccipital fasciculus, (e) inferior longitudinal fasciculus, (f) superior longitudinal fasciculus, (g) uncinate fasciculus, (h) temporal part of the superior longitudinal fasciculus, and (i) the minor and major forceps. On the left, streamlines are visualized as a summed projection over all slices for each visualized orientation. On the right, violin plots are used to depict the group differences in FA values (*x*‐axis) for each of the tests (*y*‐axis). Left (l) and right (r) hemispheres are visualized separately. The top violin (magenta) contains the results of the nonoptimal alertness group whereas the bottom violin (gray) contains the results of the optimal group. For effect size evaluation, each violin contains quartile‐lines and corresponding calculated values for Cohen's *D* and Akinshin's γ_p_ shown on the rightmost column.

**Figure:**
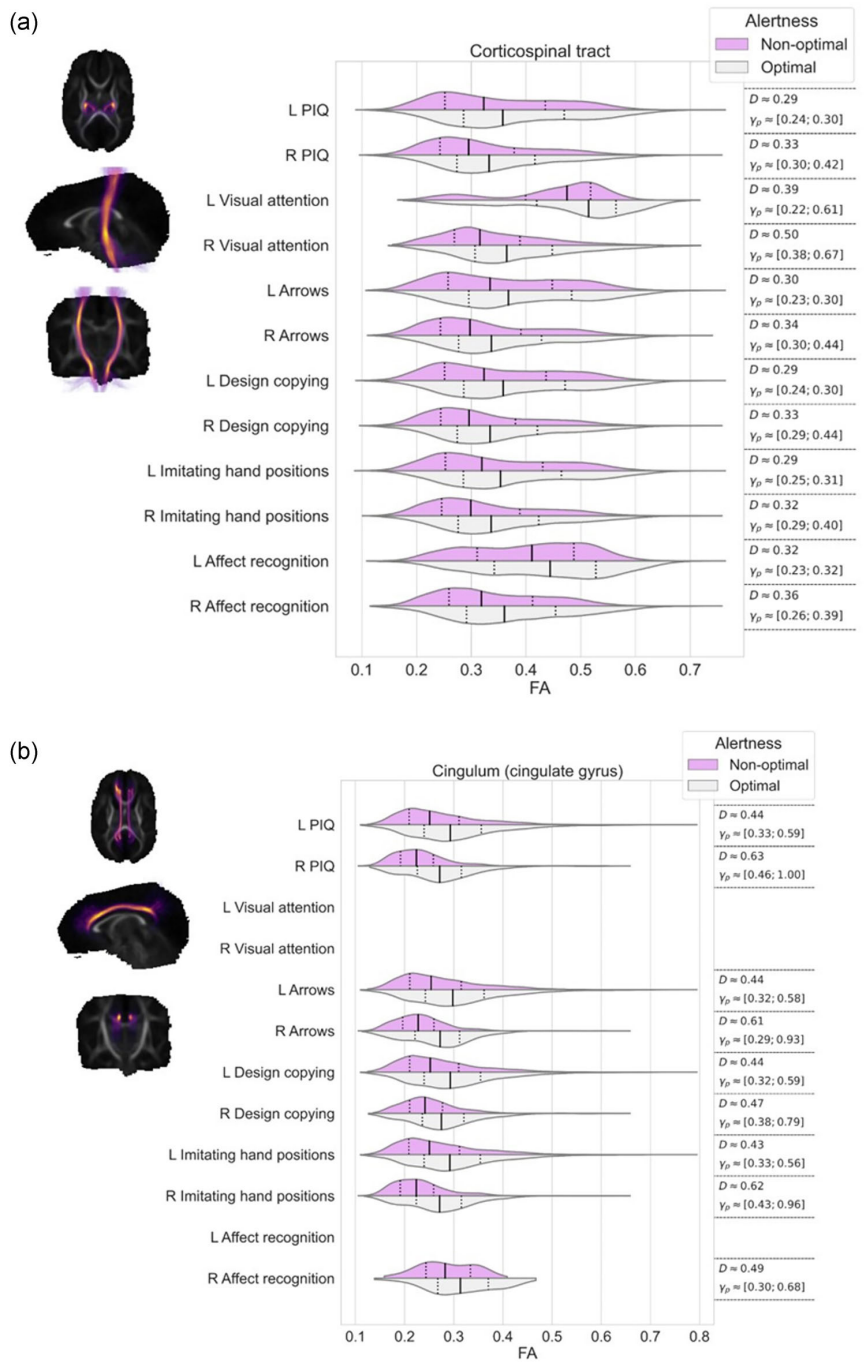

**Figure:**
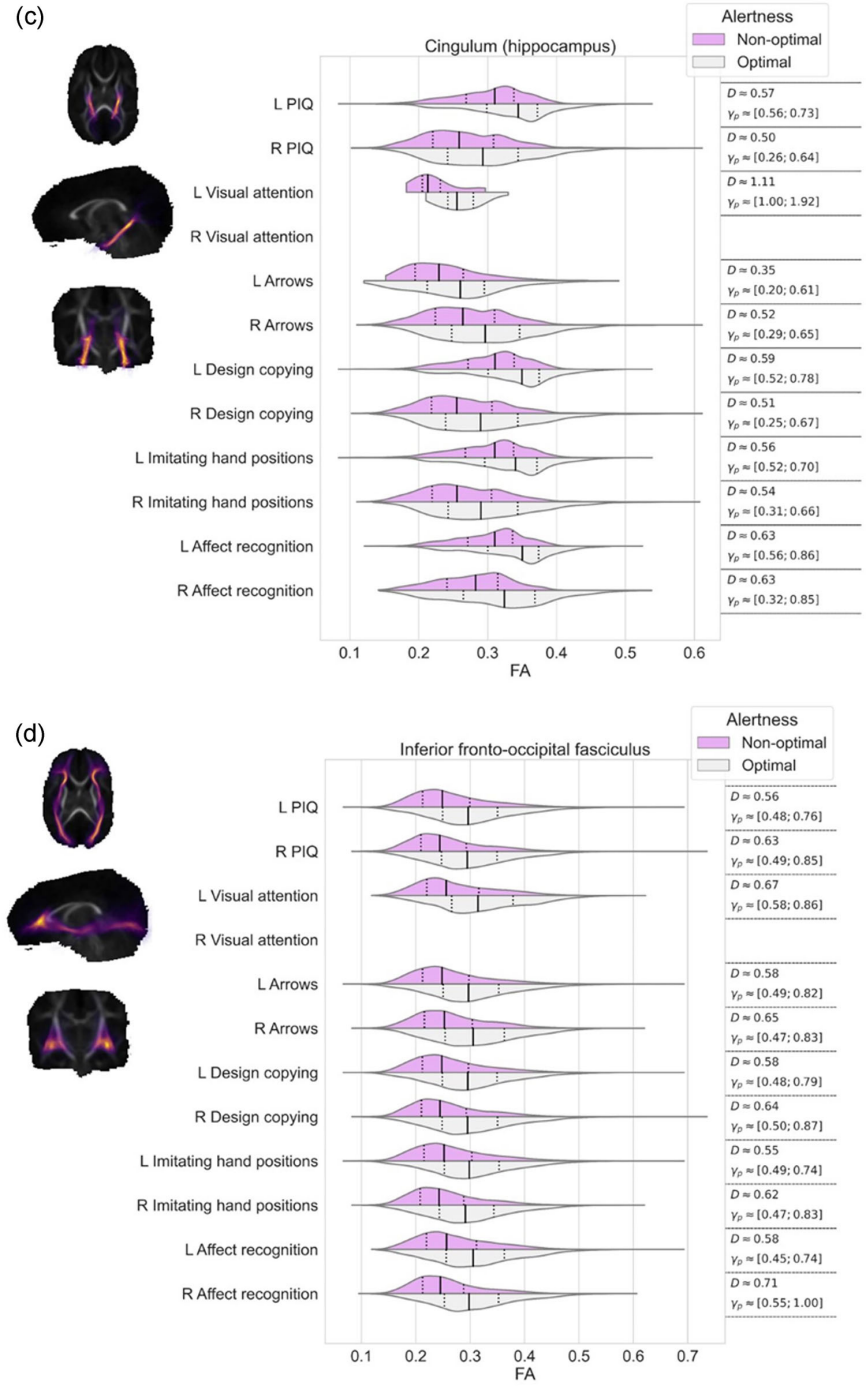

**Figure:**
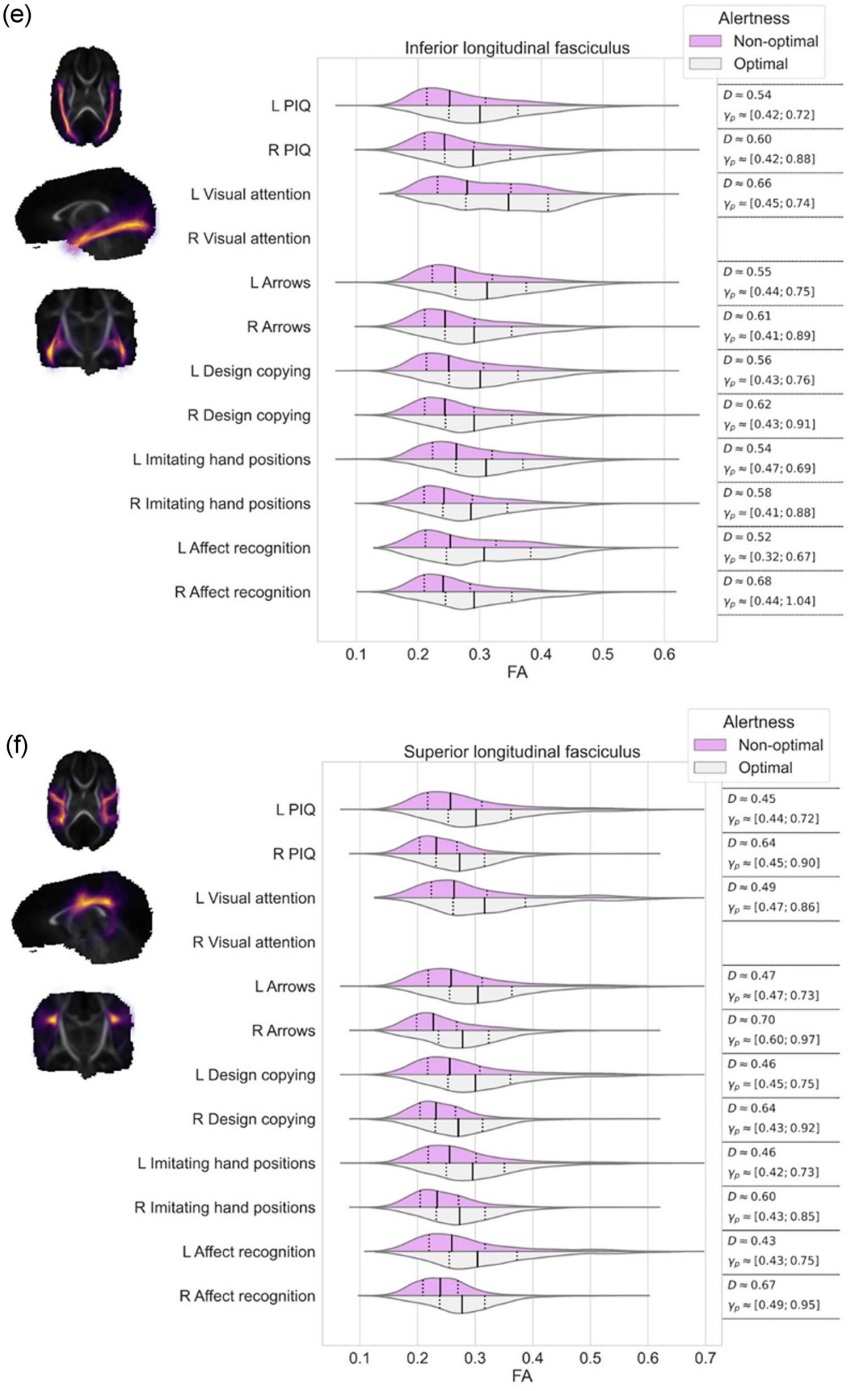

**Figure:**
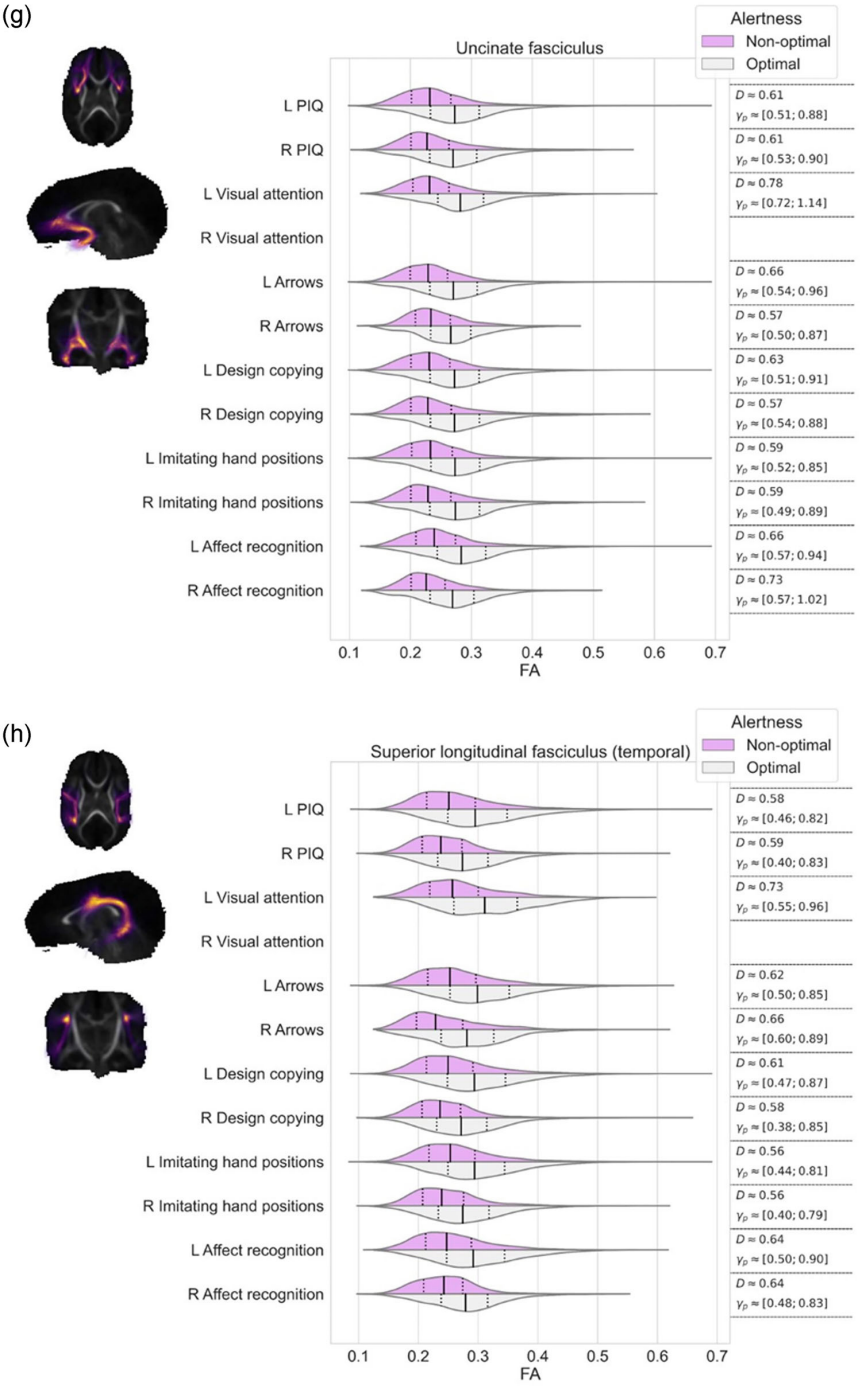

**Figure:**
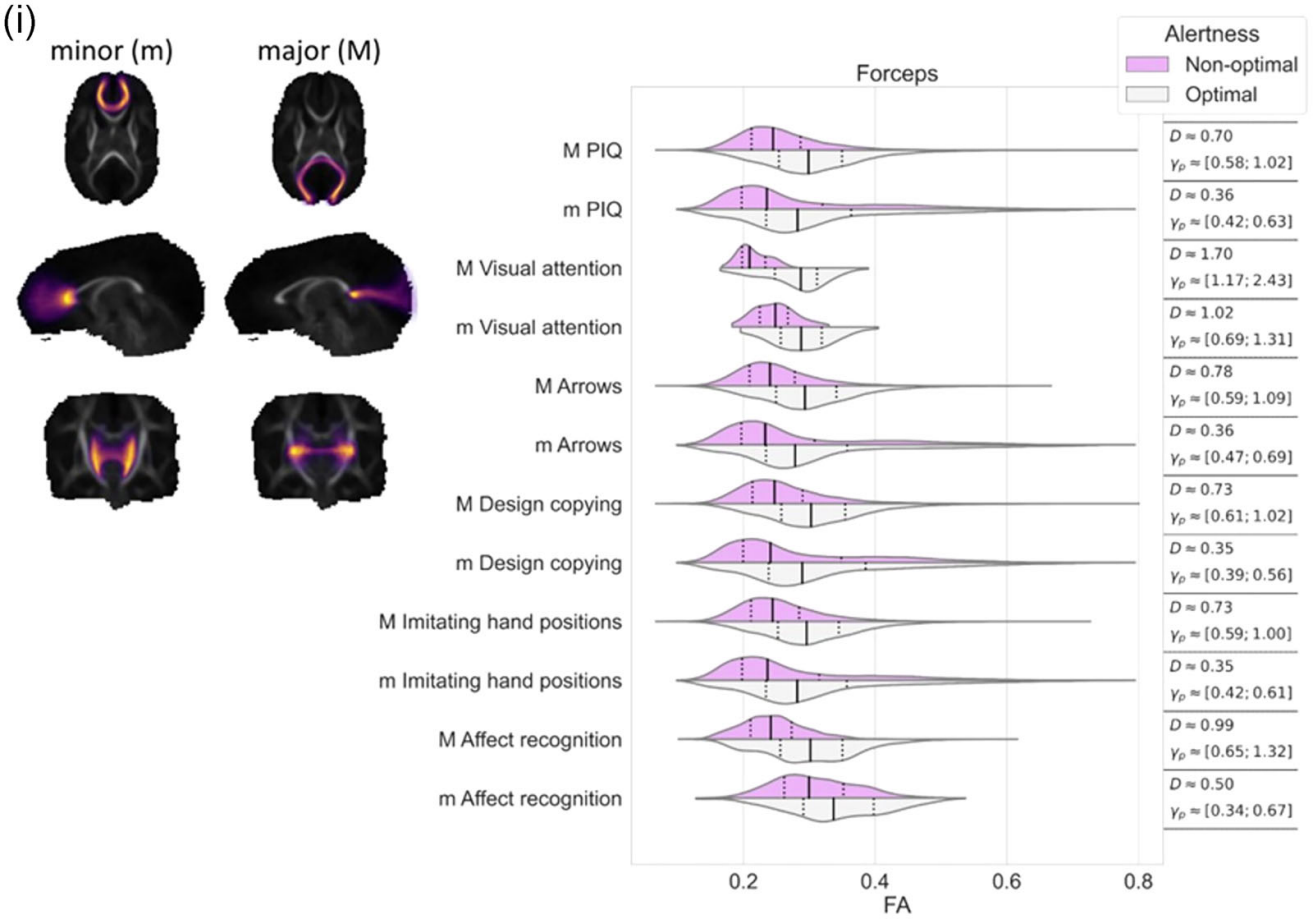

**TABLE 3:** Summary of effect sizes, that is, differences in FA values in relation to neuropsychological outcome at 6.5 years

		Perceptual intelligence quotient	Executive functioning/attention	Visuospatial processing		Sensorimotor functioning	Social perception
			Visual attention	Arrows	Design copying	Imitating hand positions	Affect recognition
**CST** (corticospinal tract)	Left	0.29	0.39	0.30	0.29	0.29	0.32
	Right	0.33	0.50	0.34	0.33	0.32	0.36
**CGY** (cingulate gyrus)	Left	0.44		0.44	0.44	0.43	
	Right	0.63		0.61	0.47	0.62	0.49
**CHI** (cinculum hippocampus)	Left	0.57	1.11	0.35	0.59	0.56	0.63
	Right	0.50		0.52	0.51	0.54	0.63
**IFOF** (inferior frontooccipital fasciculus)	Left	0.56	0.67	0.58	0.58	0.55	0.58
	Right	0.63		0.65	0.64	0.62	0.71
**ILF** (inferior longitudinal fasciculus)	Left	0.54	0.66	0.55	0.56	0.54	0.52
	Right	0.60		0.61	0.62	0.58	0.68
**SLF** (superior longitudinal fasciculus)	Left	0.45	0.49	0.47	0.46	0.46	0.43
	Right	0.64		0.70	0.64	0.60	0.67
**UF** (uncinate fasciculus)	Left	0.61	0.78	0.66	0.63	0.59	0.66
	Right	0.61		0.57	0.57	0.59	0.73
**SLF temp** (superior longitudinal fasciculus temporal part)	Left	0.58	0.73	0.62	0.61	0.56	0.64
	Right	0.59		0.66	0.58	0.56	0.64
**FMA** (forceps major)		0.70	1.7	0.78	0.73	0.73	0.99
**FMI** (forceps minor)		0.36	1.02	0.36	0.35	0.35	0.50

Cohen's *D* was used to calculate effect sizes. Cohen's criteria for small effec size is Cohen's d 0.2, for medium 0.5 and for large 0.8 or greater.

As shown in Figure 2, children with optimal visual alertness had higher FA in all examined white matter bundles than children with nonoptimal alertness. Furthermore, we showed that this higher FA measured at term age correlated with the higher standard scores in NEPSY subtests, Visual attention, Arrows, Design copying, Imitating hand positions and Affect recognition, and also with the higher PIQs at 6.5 years (nonparametric permutation test *p* < .05).

We detected the tract‐based spatial differences in FA values between the children with optimal and nonoptimal visual alertness in relation to the standard scores in Visual attention test, especially in the CHI left, FMI and FMA (Figure 2c and I and Table 3) and IFOF left, ILF left, UN left, and SLF_temp (Figure 2d, e, g, and h and Table 3).

The notable (effect size over 0.5) tract‐based spatial differences in FA values were found between the children with optimal and nonoptimal alertness in relation to PIQs in the CHI, IFOF, ILF, SLF right, UN, SLF_temp, and FMA (Figure 2c–i and Table 3), in relation to the standard scores in Arrows in the CGY right, CHI right, IFOF, ILF, SLF right, UN, SLF_temp, and FMA (Figure 2b–i and Table 3) and in relation to the standard scores in Design copying in the CHI, IFOF, ILF, SLF, UN, SLF_temp, and FMA (Figure 2c–i and Table 3).

We found the tract‐based spatial differences in FA values (effect size over 0.5) in relation to the standard scores in Imitating hand positions in the CGY right, CHI, IFOF, ILF, SLF right, UN, SLF_temp, and FMA (Figure 2b–i and Table 3) and in relation to standard scores in Affect recognition in the CHI, IFOF, ILF, SLF right, UN, SLF_temp, FMA, and FMI (Figure 2c–i and Table 3) between the children with optimal and nonoptimal alertness.

## 4 DISCUSSION

In this study, we demonstrated that optimal visual alertness at term age is related to the higher FA values in the white matter of the whole brain. In specific white matter tracts, the higher FA values are associated with better PIQ, visuospatial processing, sensorimotor function, and social perception at preschool age. Our observation supports the hypothesis that the newborn's visual alertness is an early building block for later neurocognitive and social cognitive development.

It is suggested that visual function in the newborn infant is mediated through subcortical pathways (Dubowitz et al., 1986) and the cortex starts to maturate at about 2 months postnatally (Ramenghi et al., 2010). This is based on the findings that newborns can fix their gaze and follow a stimulus even if they have severe occipital white matter injury (Dubowitz et al., 1986). Furthermore, infants with a severe lesion in the basal ganglia but intact white matter and cortex had poor visual abilities suggesting the key role of the subcortical integrity for the earliest visual function (Mercuri et al., 1997). However, Bassi et al. (2008) showed using diffusion tensor imaging a correlation between visual function and FA in the optic radiations which did not reflect a widespread white matter abnormality. They suggested that at term age the maturation and the integrity of optic radiations is important for the normal development of visual function in preterm infants. We have earlier shown in our cohort that good visual fixation with sustained alertness relates to increased FA level across the white matter with no visually apparent spatial emphasis (Stjerna et al., 2015). However, good visual orientation related to increased FA in the optic radiations (Stjerna et al., 2015). Our finding agrees with earlier studies that have shown the importance of early visual function for later visuocognitive development (Ramenghi et al., 2010; Stjerna et al., 2015). In this study, we demonstrated the structural differences in the white matter between preterm infants with optimal and nonoptimal visual alertness at term age and the relationship to neurocognitive outcome including social cognition skills at preschool age.

### 4.1 Performance IQ and visuospatial processing

We observed the association between optimal visual alertness and the higher FA values at term age especially in the FMA, visual and associative pathways and the higher PIQ and standard scores in visuospatial processing tests at preschool age. Our finding is compatible with earlier finding that the reduced splenium FA contributing the forceps major at term age was associated with abnormal neurodevelopment at 1–1/2 years of age (de Bruïne et al., 2013; Rose et al., 2009). Interestingly, Weinstein et al. (2016) reported the opposite trend, lower FA and higher radial diffusivity in the genu of the corpus callosum at term age correlated positively with hand and eye coordination at 2 years. FA changes nonlinearly with age in occipital and temporal regions, decreasing between 34 and 39 weeks followed by an increase from 40 to 43 weeks (Aeby et al., 2012). These fluctuations in FA and additional risk factors, such as perinatal neuronal injury, and the exposure to environmental stimuli at early age may explain these differences in the white matter maturation. Our study included children born before 28 gestational weeks whereas the study by Weinstein and colleagues included children born before 34 gestational weeks which may explain the different results. Among adolescents, who were born preterm and underwent MRI and cognitive assessment at 19 years, the scores in visual‐motor performance test correlated positively with the FA values in the corpus callosum, IFOF, and ATR (Sripada et al., 2015).

A general disconnection between the anterior and posterior brain region and between inter‐hemispheric tracts, especially in children with nonoptimal visual alertness, may explain the relationship between nonoptimal visual alertness and low scores on the visual perception test. Preterm children are reported to be at risk for deficit especially in visuospatial perception (Allotey et al., 2018). Our finding emphasizes the importance of the early connections of visual pathways for later visuocognition. Moreover, the abnormal maturation of the visual pathways and SLF may be a factor contributing the association between nonoptimal visual alertness and lower PIQ.

### 4.2 Executive functioning

In agreement with earlier studies, we demonstrated relationship between optimal visual alertness and higher FA values at term age and better visual attention at 6.5 years. The damage in the white matter in inferior occipital and cerebellar regions in preterm infant at term age has shown to associate with impaired executive functioning 7 years later (Thompson et al., 2014). A study of very low birth weight adolescents observed correlation between low executive functioning and low FA in the CHI and IFOF (Skranes et al., 2009). Furthermore, the better performance in the spatial working memory test, which measures the ability to retain and manipulate spatial information, is associated with higher FA in tracts including SLF, IFOF, ILF, FMA, corticospinal tracts, and corpus callosum among adolescents born preterm (Loe et al., 2018). SLF and IFOF are also related to attention regulation, one of the cornerstones of human cognition (Skranes et al., 2007). Altered microstructure of the right SLF has shown to relate to attention skills among adolescent born preterm (Frye et al., 2010).

These findings indicate a possible link between the altered white matter microstructure in inter‐ and intra‐hemispheric tracts and impaired executive functions (Loe et al., 2018; Skranes et al., 2009). Moreover, our finding indicates the importance of the early and healthy development of the structural connectome and visual abilities for the later executive functioning.

### 4.3 Sensorimotor functioning

The higher FA at term age correlated with better standard scores in Imitating hand positions test at the preschool age. Our finding agrees with earlier observations that the imitation of meaningless gestures is associated with dorso‐dorsal visuomotor stream (Achilles et al., 2019). Both IFOF and SLF white matter tracts have cortical terminations in the parietal lobe (Catani et al., 2013). They probably subserve the dorsal stream that is vulnerable to prematurity due to the combination of sensitivity to periventricular and white matter injury and premature visual experience (Atkinson, 2017). Further, the posterior fibers of corpus callosum are involved in the processing of somatosensory information by the connecting parietal, temporal, and occipital lobes and later corpus callosum seems to play an important role in cognitive functions. Nonoptimal visual alertness together with the microstructural alterations of the white matter seen already at term age seem to be associated with the difficulties in the Imitating hand positions at preschool age.

### 4.4 Social perception

We found the association between FA values at term age and the performance in Affect recognition test, assessing emotions from facial expressions, at preschool age. FMA is involved in the processing of visual cues, and CHI is part of the limbic system, and it connects the amygdala to cortex (Catani et al., 2013). Altered CHI structure in the preterm brain at near‐term age is associated with impaired social‐emotional development at 18–22 months (Lee et al., 2021). We demonstrated that lower FA in the CHI relates with impaired Affect recognition also at preschool age. Amygdala takes part in orientating the infant's attention toward faces (Leppänen & Nelson, 2006), and newborn's preferential attention to human faces and eyes immediately after birth plays a crucial role in early social and cognitive development (Hoehl et al., 2009). Moreover, the reduced FA value in the ILF and IFOF correlate with impaired performance in face processing tasks and sudden disruption of the ILF has been associated with neuropsychological impairments of visual cognition and socioemotional (Herbet et al., 2018; Thomas et al., 2009). The ILF connects the visual cortex and the amygdala and the disruption of the amygdala‐occipital pathways may be the pathopsychological basis of impaired emotional processing (Herbet et al., 2018). The ILF maturates earlier and faster than associative white matter tracts (e.g., the cingulum and UN) (Dubois et al., 2014), thus alterations in the ILF white matter maturation are likely to have influence on some basic cognitive and perceptual processes. Very low birth weight children have difficulty in interpreting the emotions of others, mainly due to a failure to identify key nonverbal cues from facial and body movements, which suggested that the difficulties primarily reflect a visual processing deficit (Williamson & Jakobson, 2014). In agreement with earlier studies, we demonstrated that the microstructural alterations in visual pathways (ILF and IFOF) may have influence on the development of social skills and these alterations may be the pathopsychological basis of deficit in a visual processing leading to the failure to identify nonverbal cues. Moreover, the alteration in ILF may influence to amygdala‐occipital pathways, which have important role in orientating the infant's attention toward faces (Leppänen & Nelson, 2006). The sensory exposures in the neonatal intensive care unit may modify especially the maturation of these sensory pathways. Our finding suggests that the integrity of CHI and FMA in addition to visual pathways have an important role for the development of social skills.

### 4.5 The reliability of dMRI and FA estimates

**FIGURE 3:**
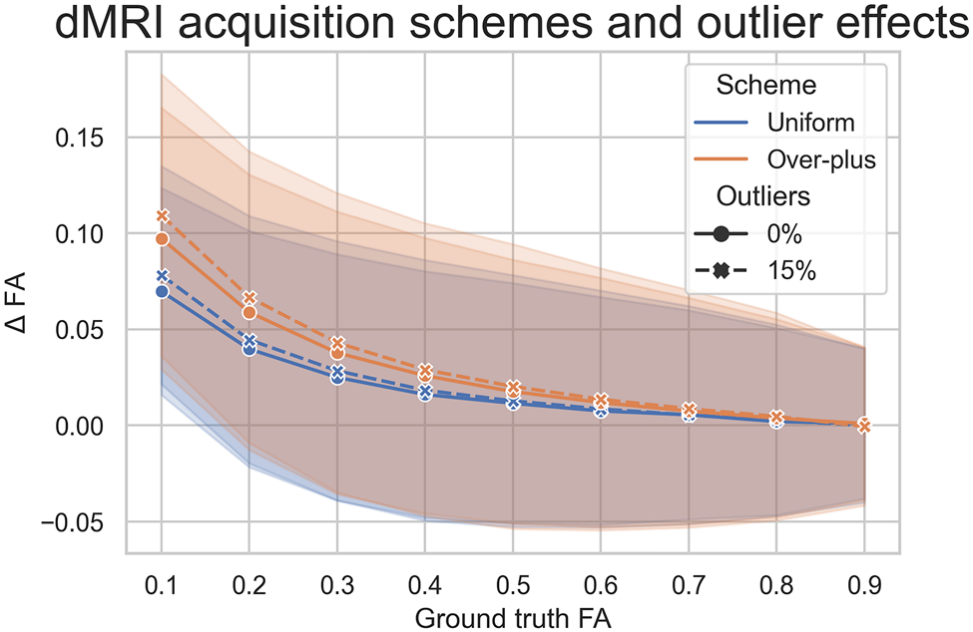
Simulated evaluation of noise and outlier effects on the used over‐plus gradient scheme and comparison to the current “gold‐standard” uniform gradient scheme. The *x*‐axis depicts the ground truth FA values used in the simulation and the *y*‐axis depicts the deviation (ΔFA) from those ground truth values. The average deviation is shown with the solid (0% outliers) and dashed (15% outliers) lines and the blue (uniform) and orange (over‐plus) areas depict the corresponding standard deviations. The effect of noise is very prominent with lower FA values leading to distinct over estimation of FA in all cases. As all subjects in our study were scanned using the same over‐plus scheme, they all are affected by the same systematic measurement error. Therefore, the largest average error (the difference between solid and dashed orange lines) due to these effects in our study is approximately 0.01 at low FA values diminishing to near zero at higher FA values. The difference between blue and orange lines could be useful when comparing our results to studies that have used uniform sampling scheme.

We investigated possibilities to perform diffusion tensor and constrained spherical deconvolution‐based probabilistic and deterministic tractography methods (Maier‐Hein et al., 2017) and deemed that this data acquired using over‐plus gradient scheme was not suitable for such analysis. We evaluated the reliability of FA values derived from the used gradient scheme in the presence of outliers with Monte–Carlo simulations. The results of Monte–Carlo simulations (Figure 3) demonstrate that the redundancy obtained with the three repetitions helped to obtain reliable FA estimates even in the case of 15% of outliers, which was the largest number of outliers in any of the datasets. Our simple one‐voxel Monte–Carlo simulation does not capture all the fine details of human brain microstructure. However, it provides the necessary information for us to know how much we can trust our FA estimates. Combined with the spatial information from the JHU DTI atlas, we were also able to approximate which white matter structures these reliable FA estimates belong to without needing to perform manual brain segmentations.

### 4.6 Limitations

The main limitation of this study is the relatively small number of EPT born children who had undergone all tests including dMRI and the lack of control subjects. There were more males in our study cohort. It has been reported that the higher incidence of abnormal neurodevelopment in preterm males is associated with lower FA at least in splenium and right posterior limb of internal capsule (Rose et al., 2009). We took this into account in the TBSS analysis and used sex as a covariate factor. Therefore, it is unlikely that this would affect the detected relationship between visual alertness, FA values at term age and neurocognitive outcome at preschool age. The study cohort included EPT children limiting us to make conclusion only on EPT children.

## 5 CONCLUSION

This study shows neuroanatomically specific regional differences in the FA values between EPT children with optimal and nonoptimal visual alertness at term age and the relation to neurocognitive functioning and social cognition skills at 6.5 years. Our results provide neurobiological evidence for the importance of the early visual abilities for later neurocognitive and social cognitive development. Visual alertness is likely a powerful index of the maturation and connectivity of white matter and especially visual pathways, and visual alertness seems to relate to the microstructural changes as measured by dMRI. Our observations support the hypothesis that newborn's visual alertness is likely an early building block in the upcoming cascades of visuocognitive, sensorimotor, and social cognition development, and thus visual alertness could be used as a predictor of later neurocognitive functions. The observed spatial differences in the white matter maturation at term age could be used to develop individualized interventions and for benchmarking early therapeutic interventions.

## CONFLICT OF INTEREST STATEMENT

The authors declare that the research was conducted in the absence of any commercial or financial relationships that could be construed as a potential conflict of interest.

### PEER REVIEW

The peer review history for this article is available at https://publons.com/publon/10.1002/brb3.3048.

## Data Availability

The data that supports the findings of this study is available from the corresponding author upon reasonable request.
